# Study of the role of transmembrane emp24 domain-containing protein 2 in oral squamous cell carcinoma

**DOI:** 10.1590/1678-7757-2024-0305

**Published:** 2025-01-27

**Authors:** RUAN Zhao-wei, JIAO Xue-feng, XIAO Gao-tian, CHEN Jin-ling, LI Jun, LV Shu-ying

**Affiliations:** 1 Ningde Normal University Department of Stomatology Fujian China Ningde Hospital Affiliated to Ningde Normal University, Department of Stomatology, Fujian, China.; 2 Fujian Medical Universtity Stomatology Center Fujian China The First Affiliated Hospital of Fujian Medical Universtity, Stomatology Center, Fujian, China

**Keywords:** Oral squamous cell carcinoma, TMED2, ARF1, ERK

## Abstract

**Objective:**

This study aimed to investigate the role of transmembrane emp24 domain-containing protein 2 (TMED2) in oral squamous cell carcinoma (OSCC).

**Methodology:**

A bioinformatics analysis was first conducted to explore TMED2 expression in OSCC and its relation with overall survival. The analysis results were further verified by assessing TMED2 expression levels in human normal oral keratinocyte cells and human OSCC cell lines using quantitative real-time polymerase chain reaction and the Western blot. Finally, the effects of TMED2 knockdown and overexpression on the expression levels of TMED2, ADP-ribosylation factor 1, extracellular signal-regulated kinase ½, and phospho-extracellular signal-regulated kinase ½ proteins were examined in cells using the Western blot.

**Results:**

The GEPIA2 database showed that OSCC tissues expressed more TMED2 than normal tissues. At the cellular level, TMED2 expression significantly increased in SCC-4, HSC-3, and CAL-27 cells than in human normal oral keratinocyte cells. TMED2 knockdown reduced cell proliferation, increased the apoptosis rate in SCC-4 cells, and led to a higher proportion of cells in the G0/G1 phase and a lower proportion in the S phase.

**Conclusion:**

TMED2 may promote OSCC cell proliferation and inhibit apoptosis, potentially by activation of the ADP-ribosylation factor 1/ extracellular signal-regulated kinase ½ signaling pathway.

## Graphical abstract

**Figure:**
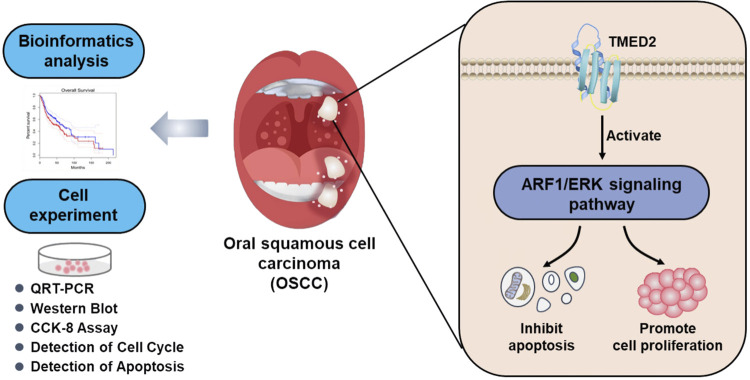

## Introduction

Oral squamous cell carcinoma (OSCC) accounts for more than 90% of all head and neck malignancies, occurring more often than any of them.^1,2^ It has emerged as a serious public health problem, among the top eight cancers in global mortality.^3^ Surgery remains the cornerstone treatment for OSCC, typically supplemented by radiotherapy and chemotherapy. Recent advances in treatment have improved survival rates and quality of life for OSCC patients, particularly those undergoing surgical interventions.^4^ Despite these advances, challenges remain and also the urgent need to find new effective targets to further improve survival outcomes in OSCC patients.

Genetic alterations are associated with the development and progression of OSCC, such as the altered activity of p53,^5^ mutations of Notch,^6^ and down-regulation of N-myc downstream regulated gene 2 expression.^7^ This strong correlation underscores the importance of investigating specific genes to enhance the treatment and drug development of OSCC. Transmembrane emp24 domain-containing protein 2 (TEMD2) belongs to the p24 receptor family, being mainly involved in vesicular trafficking of proteins between the endoplasmic reticulum and Golgi apparatus.^8^ Previous studies have established a significant link between TMED2 and cancer progression. Shi-Peng, et al.^9^ (2017) found TMED2 as an oncogene in ovarian cancer, promoting cancer by activating the insulin-like growth factor 2/insulin-like growth factor 1 receptor/phosphoinositide 3-kinase/protein kinase B pathway. Similarly, Ge, et al.^10^ (2020) have reported high expression of TMED2 mRNA in multiple myeloma cell lines and shown that TMED2 promotes cell proliferation and inhibits apoptosis by regulating caspase 3/7 activity. However, the role of TMED2 in OSCC cells and its associated mechanisms remain unclear and require further investigation.

ADP-ribosylation factor 1 (ARF1) is a Golgi-associated GTPase that recruits a variety of effector proteins on the Golgi apparatus, including coat proteins to form vesicles and lipid-modifying enzymes, and is an important factor in maintaining the structure, morphology, and function of the Golgi apparatus during cell mitosis.^11^ ARF1 is highly expressed in various malignancies and plays a significant role in tumor initiation, progression, and metastasis.^12-14^ Extracellular signal-regulated kinase (ERK), specifically extracellular signal-regulated kinase 1/2, belongs to the mitogen-activated protein kinase family. ERK1/2 has the physiological function of transmitting extracellular signals to intracellular targets and is integral to cell proliferation, differentiation, and stress response [14]. Luchsinger, et al.^12^ (2018) have suggested that ARF1 may promote cell proliferation and inhibit apoptosis by activating the ERK pathway in MDA-MB-231 breast cancer cells. He, et al.^13^ (2019) found that Trichostatin A suppresses the migration and invasion of head and neck squamous cell carcinoma cells by inhibiting epidermal growth factor receptor/ADP-ribosylation factor 1 signaling. Moreover, ARF1 can activate AKT and ERK signaling in multiple myeloma cells and induce cell adhesion-mediated drug resistance.^14^ ARF1 promotes colon tumorigenesis, representing a promising biomarker and therapeutic target in colorectal cancer.^15^ Exo2, a small-molecule inhibitor that reduces ARF1 activation, effectively suppresses prostate cancer cell proliferation by blocking ERK1/2 activation.^16^ Previous studies have reported the oncogenic role of TMED2 in some cancers, including hepatocellular carcinoma, prostate cancer, and colorectal cancer.^17,18^ However, the potential activation of ARF1/ERK signaling by TMED2 remains speculative.

Therefore, this study aimed to explore the role of TMED2 in OSCC and its underlying mechanism. First, this study analyzed TMED2 expression in OSCC cells and its relation with the clinical prognosis of OSCC patients by combining bioinformatics techniques, the Western blot, and quantitative real-time polymerase chain reaction (qRT-PCR). Furthermore, the cell counting kit-8 assay and flow cytometry served to analyze the effects of TMED2 modulation (both knockdown and overexpression) on cell proliferation, apoptosis rate, and cell cycle. Additionally, this research used the Western blot to detect ARF1/ERK signaling pathway-related protein expression in cells.

## Methodology

### Bioinformatics analysis

The GEPIA2 database (http://gepia2.cancer-pku.cn/#index) was searched for the expression level of TMED2 in OSCC tissues and its correlation with the overall survival (OS) rate of OSCC patients.^19^ Specifically, TCGA-OSCC data were accessed and selected within the GEPIA2 interface to ensure consistency and specificity to the OSCC cohort. Next, the box plot function in GEPIA2 was employed to visualize the expression levels of genes of interest in TCGA-OSCC samples when compared to normal controls. Finally, the GEPIA2 Kaplan-Meier plotter was used to assess the correlation between gene expression levels and patient survival outcomes in the TCGA-OSCC cohort.

### Cell culture and transfection

Human OSCC cell lines SCC-4, HSC-3, and CAL-27 were often used to explore the pathological mechanism of OSCC.^20^ In this study, human normal oral keratinocytes (HOK) cells (SC-2610) were obtained from Sciencell (Carlsbad, USA), SCC-4 (ATCC^®^ CRL-1624™) and CAL-27 (ATCC^®^ CRL-2095™) were purchased from ATCC cell bank (Manassas, VA, USA), and HSC-3 cells (JCRB 0623) were purchased from the Osaka National Institute of Health Sciences (Osaka, Japan). All cell lines were cultured in a DMEM medium (#11965092, Gibco, USA) supplemented with 10% fetal bovine serum (FBS, # 10099141, Gibco, USA), 100 IU/mL penicillin, and 100 μg/mL streptomycin (#P1400, Solarbio, China), and placed in an incubator at 37 °C with 5% CO_2_.^21^

SCC-4 and HSC-3 cells in the logarithmic growth phase were digested, resuspended, and seeded in 6-well plates to reach 80% – 90% confluence. Then, SCC-4 cells were transfected with TMED2 shRNA (sh-TMED2 group) and negative control shRNA (sh-NC group); HSC-3 cells were transfected with pcDNA-TMED2 (TMED2 group) and pcDNA3.1 vector (mock group), respectively. TMED2 shRNA, negative control shRNA, pcDNA-TMED2, and pcDNA3.1 vector were designed and synthesized by Shanghai GenePharma Co., Ltd. The sequences of TMED2 shRNA was 5’-AGGACCAGATAACAAAGGAAT-3’.

Transfection was carried out according to the instructions of Lipofectamine^TM^ 2000 (#11668019, Thermo Fisher Scientific, USA). The culture medium was aspirated from the dish and the cells were washed once with PBS. The plasmids and lipo2000 were diluted with 200 μL opti-MEM, mixed gently, and allowed to stand at room temperature for 20 min. The transfection reagent was added to each dish and, after six hours of incubation, the medium was replaced with fresh DMEM containing 10% FBS. The transfected cells were collected after another 48-hour culture at 37 °C with 5% CO_2._

### QRT-PCR

Total cellular RNA was extracted with the Trizol reagent (#T9424, Sigma, USA) and quantified using Nanodrop. The extracted RNA was reverse transcribed into cDNA using the SuperScript VILO cDNA Synthesis Kit (#11754050, Invitrogen, USA). Subsequently, qRT-PCR was performed on a Thermal Cycler Dice^®^ Real Time System utilizing SYBR^®^ Premix Ex Taq™ II (#RR820, Takara, Japan). Data were analyzed using the 2^-^ method with GAPDH serving as the internal control. The primer sequences used are shown in Table 1.

**TABLE 1:** Primer sequences used in the qRT-PCR

Gene	Primer sequence (5’ to 3’)
TMED2	F: CAGCCGTAAAGCACGAACAGGA
	R: GTCATGGCAACTAGAACAAGAGC
GAPDH	F: GTCTCCTCTGACTTCAACAGCG
	R: ACCACCCTGTTGCTGTAGCCAA

### Western blot

Total cell protein was extracted by RIPA lysate (#R0010, Solarbio, China) on ice, and its concentration was determined using the BCA Protein Assay Kit (#23225, Thermo, USA). Proteins (10/20 µg) were separated on 10 or 12% sodium dodecyl sulfate polyacrylamide gel electrophoresis (SDS-PAGE) and transferred to PVDF membranes, which were then blocked with 5% skim milk. Following blocking, the membranes were subjected to two rounds of incubation: an overnight incubation at 4 °C with primary antibodies (TMED2, #11981-1-AP; ARF1, #10790-1-AP; ERK1/2, #11257-1-AP; p-ERK1/2, #28733-1-AP; β-actin, #66009-1-Ig; Proteintech, USA) and a one-hour incubation at room temperature with secondary antibodies. The membranes were washed three times after each incubation. The enhanced chemiluminescence solution (#PE0010, Solarbio, China) was applied for detection, and images were captured using GE Healthcare imaging equipment. The Image J software was used to analyze the intensity of protein bands, with β-actin serving as the internal reference to calculate the relative expression of the proteins.

### CCK-8 assay

Cells were seeded into 96-well plates at a density of 2 × 10^3^ cells/well and cultured for 48 hours. Subsequently, 10 μL of the CCK-8 solution (#C0037, Beyotime, China) were added to each well, followed by a further incubation of 2.5 hours. Absorbance at 450 nm was then measured with a microplate reader.

### Detection of cell cycle

Cells were digested with trypsin and centrifuged at 300 × g for 5 min and then washed twice with PBS. Subsequently, the cells were fixed in 75% ethanol overnight at −20 °C. After fixation, the cells were washed again twice with PBS and treated with RNase to remove RNA. Then 500 μL of the PI staining solution were added to the cells for a 15-min incubation at room temperature in the dark. Finally, cell cycle analysis was conducted using the FACScan system.

### Detection of apoptosis

The Annexin V-FITC/PI Apoptosis Detection Kit (#CA1020, Solarbio, China) was employed to detect apoptosis after a 48-hour transfection. Specifically, cells were incubated with FITC-Annexin V and PI for 15 min at room temperature in the dark, and the apoptosis rate was determined using a FACScan system within one hour.

### Statistical analysis

Statistical analysis was performed using SPSS 22.0 and Graphpad prism 9 for graphical presentations. All data are shown as mean ± standard deviation (SD). Before conducting the statistical tests, the normality of data distribution was assessed using the Shapiro-Wilk test, and homogeneity of variance was evaluated using Levene’s test. Differences between the two groups were analyzed using the independent t-test, whereas comparisons between multiple groups were performed using one-way analysis of variance (ANOVA) followed by LSD-t test for pairwise comparisons. The value of *P*<0.05 was considered statistically significant.

## Results

### TMED2 is highly expressed in OSCC tissues and cells.

**Figure 1:**
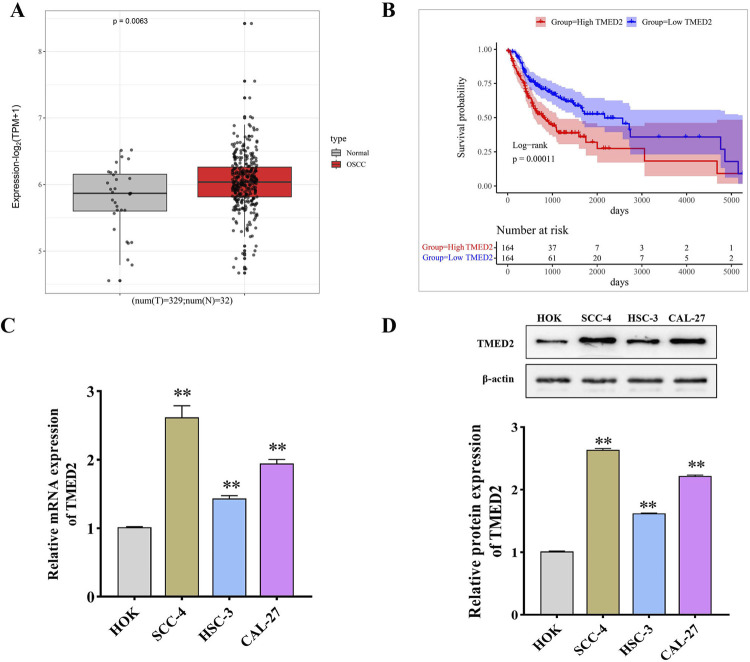
TMED2 is highly expressed in OSCC tissues and cells. A: GEPIA2 database-based analysis of TMED2 expression in head and neck squamous cell carcinoma; B: GEPIA2 database-based analysis of the OS curve of TMED2 in OSCC patients; C: qRT-PCR was used to detect the relative expression level of TMED2 mRNA in HOK, SCC-4, HSC-3, and CAL-27 cells (n=3); D: the Western blot was used to measure the relative expression level of TMED2 protein in HOK, SCC-4, HSC-3, and CAL-27 cells (n=3). **P<0.01 vs. HOK group.

To explore the association between TMED2 and OSCC development, we first analyzed the expression of TMED2 in OSCC and its relation with patients’ overall survival (OS) using the GEPIA2 database. The comparison with normal oral tissues showed an increase of TMED2 expression in OSCC tissues in the analysis result (Figure 1A). Moreover, patients with higher levels of TMED2 expression had shorter OS than those with lower TMED2 expression (Figure 1B). To strengthen the bioinformatics results, we examined the mRNA and protein expression of TMED2 in the HOK cell line and three human OSCC cell lines (SCC-4, HSC-3, and CAL-27) using qRT-PCR and the Western blot. Results showed that OSCC cells had significantly higher mRNA and TMED2 protein expression than HOK cells (*P*<0.01) (Figure 1C/D). Collectively, results suggested that TMED2 was possibly associated with the development and progression of OSCC.

### TMED2 promotes proliferation and inhibits apoptosis in OSCC cells

To explore the role of TMED2 in OSCC, we transfected SCC-4 cells with sh-TMED2 to knockdown and HSC-3 cells with pcDNA-TMED2 to induce overexpression. The following CCK-8 assay and flow cytometry analyzed the effect of altering TMED2 expression on OSCC cell dynamics.

**Figure 2:**
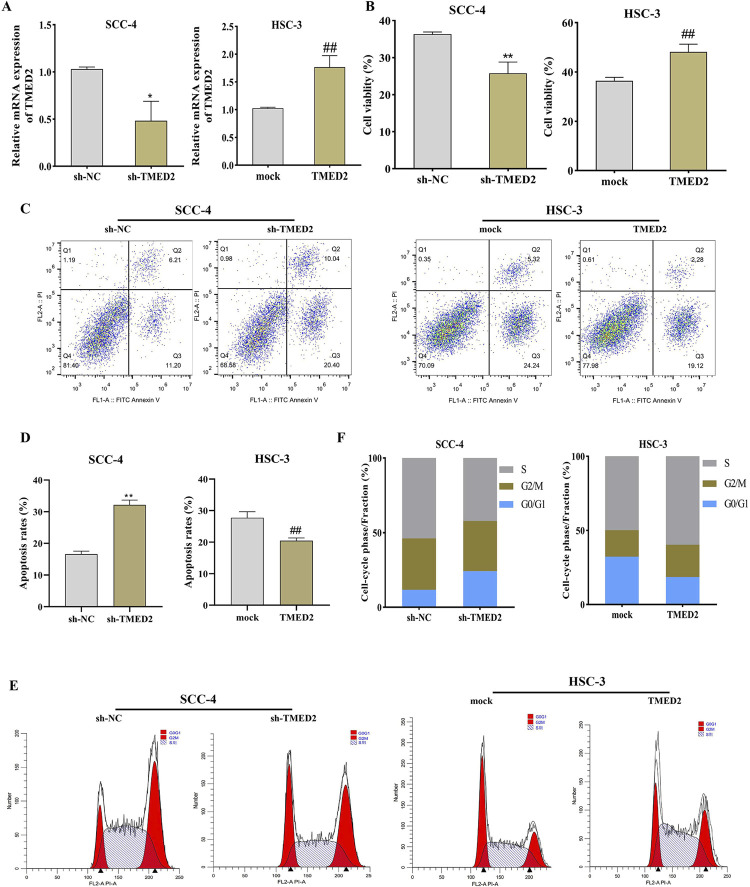
TMED2 Promotes Proliferation and Inhibits Apoptosis in OSCC cells. A: qRT-PCR was used to detect TMED2 expression in SCC-4 and HSC-3 cells transfected with sh-TMED2 and pcDNA-TMED2 (n=3); B: CCK-8 assay was used to analyze the effect of knockdown or overexpression of TMED2 on the proliferation of SCC-4 and HSC-3 cells (n=3); C-D: Flow cytometry was used to analyze the effect of knockdown or overexpression of TMED2 on the apoptosis of SCC-4 and HSC-3 cells (n=3); E-F: Flow cytometry was applied to analyze the effect of knockdown or overexpression of TMED2 on the cell cycle of SCC-4 and HSC-3 cells (n=3). *P<0.05, **P<0.01 vs. sh-NC; ##P<0.01 vs. mock.

QRT-PCR results confirmed the successful transfection, with TMED2 mRNA expression downregulated in SCC-4 cells and upregulated in HSC-3 cells (Figure 2A). Post-transfection, the cell viability of SCC-4 cells in sh-TMED2 (25.81%) decreased when compared with sh-NC (36.37%) (*P* < 0.01), whereas TMED2 overexpression in HSC-3 cells increased cell viability from 36.41 to 48.15% (*P*<0.01) (Figure 2B). The apoptosis assay showed a marked increase in apoptosis rate in SCC-4 cells after TMED2 knockdown, whereas overexpression of TMED2 in HSC-3 cells decreased the apoptosis rate (Figure 2C-D). Meanwhile, the knockdown of TMED2 increased the proportion of SCC-4 cells in the G0/G1 phase and decreased that of cells in the S phase. Conversely, overexpression of TMED2 increased the proportion of cells in the S phase and decreased the proportion of cells in the G0/G1 phase in HSC-3 cells (Figure 2E-F). These results implied that TMED2 promoted proliferation and inhibited apoptosis in OSCC cells, highlighting its potential as a therapeutic target in OSCC management.

### Knockdown of TMED2 inhibits ARF1/ERK signaling in OSCC cells

**Figure 3:**
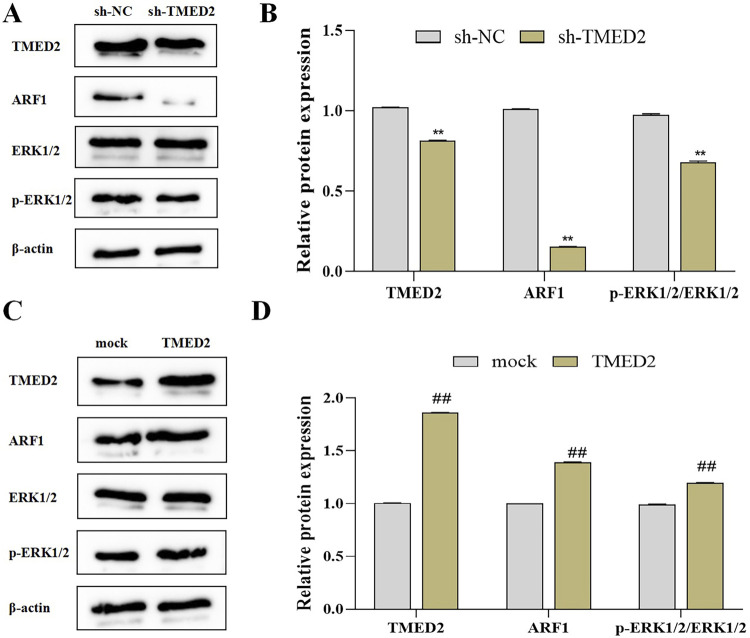
Knockdown of TMED2 suppresses ARF1/ERK signaling in OSCC cells. A-D: the Western blot was used to detect the effect of knockdown (A/B, SCC-4 cells) or overexpression (C/D, HSC-3 cells) of TMED2 on the expression levels of TMED2 and ARF1/ERK signaling proteins (ARF1, p-ERK1/2, ERK1/2) in OSCC cells (n=3).

ARF1 is a key regulator of proliferation in multiple tumors and contributes to the migration and invasion of head and neck squamous cell carcinoma.^12^ However, whether ARF1 signaling is involved in promoting OSCC cell proliferation via TMED2 remains unknown. To explore the role of ARF1/ERK in this context, we examined the expression levels of related proteins in OSCC cells following TMED2 knockdown/overexpression using the Western blot. Results showed that TMED2 knockdown in SCC-4 cells decreased expression of both TMED2 and ARF1 protein, along with a significant reduction in the ratio of p-ERK1/2/ERK1/2 (*P*<0.01) (Figure 3A/B). By contrast, TMED2 overexpression in HSC-3 cells significantly increased TMED2 and ARF1 protein levels and the p-ERK1/2/ERK1/2 ratio (*P*<0.01) (Figure 3C/D). These findings suggest that TMED2 might affect OSCC cell proliferation by activating ARF1/ERK signaling, suggesting a potential molecular target for the therapeutic intervention in OSCC.

## Discussion

OSCC is a prevalent malignant tumor in the oral cavity, with squamous cell carcinoma being its most common pathological type.^3,22,23^ Despite advances in the treatment of OSCC, multiple challenges underscore the urgent need for new effective therapeutic targets. TMED2 occurs in many organelles, including the Golgi apparatus and the endoplasmic reticulum, in which it regulates the transport of cargo proteins such as anterior gradient 2, glucagon receptor, and Gas1p.^24^ Abnormal expression of TMED2 may disrupt protein trafficking in organelles, leading to cancer progression. Previous studies have reported the oncogenic role of TMED2 in some cancers, including hepatocellular carcinoma and prostate cancer.^17^ For example, Zheng, et al.^25^ (2016) reported the significant upregulation of TMED2 in hepatocellular carcinoma, correlating it with poor clinical outcomes, whereas Vainio, et al. (2012) identified TMED3 as a potential drug target for prostate cancer.^24^

This study investigated TMED2 expression in OSCC and its association with OS using the GEPIA2 database. Our findings indicated that OSCC tissues highly expressed TMED2 in association with shorter OS in OSCC patients. Additionally, this research found elevated TMED2 levels in three human OSCC cell lines. TMED2 knockdown decreased cell proliferation, promoted apoptosis, and reduced the proportion of SCC-4 cells in the S phase, whereas TMED2 overexpression in HSC3 cells produced the opposite effects. These results indicate that TMED2 might affect the expression of some cyclins involved in cell cycle progression, although further experimental validation is required.

It is important to note that a recent study analyzing TMED2 expression in head and neck squamous cell carcinoma has similarly found that TMED2 overexpression was associated with poor prognoses.^26^ This study supports our bioinformatics findings and highlights the broader role of TMED2 in squamous cell carcinomas. However, while our study focuses on OSCC, the molecular mechanisms governing TMED2 function in different cancer types, such as head and neck squamous cell carcinoma, may vary due to distinct microenvironments and signaling pathways. Therefore, it is necessary to fully understand the role of TMED2 across different types of cancer.

Interestingly, contrasting results have been reported in other cancer types. For instance, Mishra, et al.^18^ (2019) found that TMED2 expression was significantly down-regulated in colorectal cancer, in which TMED2 acted as a metastasis suppressor, inhibiting the progression of the disease. This highlights the complex, context-dependent roles of TMED2, with tumor-promoting effects in OSCC and tumor-suppressive effects in colorectal cancer. These findings underscore the importance of investigating the function of TMED2 across various types of cancer to understand the underlying mechanisms that determine its oncogenic or tumor-suppressive roles.

This study focused on the ARF1/ERK signaling pathway, which is crucial in the progression of various cancers. ARF1 has been shown to promote proliferation and migration in ovarian cancer^27^ and activate the mitogen-activated protein kinase pathway in prostate tumors.^28^ ERK1/2, a member of the mitogen-activated protein kinase family, transmits extracellular signals to intracellular targets and is closely related to cellular proliferation, differentiation, and stress response.^29^ Prior studies, such as Haines, Schlienger, Claing^30^ (2015), have shown that ARF1 could activate ERK1/2 and promote apoptosis in triple-negative breast cancer. Similarly, the ARF1/ERK pathway has been identified as a key regulator for cancer development in other tumor types.^31^ However, while these studies have explored the role of ARF1 and ERK signaling in other cancers, the involvement of TMED2 in modulating this pathway in OSCC has been scarcely investigated. Our study fills this gap by showing that TMED2 knockdown significantly reduced ARF1 expression and modulated the p-ERK1/2/ERK1/2 ratio in SCC-3 cells, suggesting that TMED2 influences OSCC progression by this pathway. Interestingly, we observed that the knockdown of TMED2 had a more pronounced effect on ARF1 than on p-ERK, possibly due to the regulation of ERK via multiple pathways in cancer cells.

However, it is important to recognize the limitations of this study. First, while we observed clear trends in the effects of TMED2 on OSCC cell proliferation and apoptosis, we conducted experiments *in vitro*, thus constituting only an initial exploration of the role of TMED2. Further studies including *in vivo* models and additional molecular investigations are necessary to validate these findings. Second, we ignored transfection efficiency using a reporter gene (e.g., GFP), which could potentially affect the accuracy of our flow cytometry results. Future studies should incorporate this assessment for more precise evaluations. Third, since TMED2 is involved in Golgi function, performing side scatter analysis could provide insights into the effects of TMED2 on intracellular vesicles. Lastly, we neither measured ARF1 activity directly nor used specific inhibitors or activators. Incorporating these tools will help explain the mechanistic role of ARF1 in TMED2-mediated effects. Furthermore, the lack of irradiation experiments limits the clinical relevance of our findings since radiotherapy is a common OSCC treatment. Future work should investigate the response of TMED2 to radiation.

Thus, our results indicated that TMED2 promoted OSCC cell proliferation and inhibited apoptosis, potentially by activating ARF1/ERK signaling. However, the mechanisms by which TMED2 activates this signaling pathway and its subsequent effects on OSCC proliferation and apoptosis are yet to be fully explored.

## Conclusions

In summary, TMED2 is significantly higher expressed in OSCC tissue and cells (in association with poorer OS in OSCC patients). The knockdown of TMED2 significantly inhibited OSCC cell proliferation, promoted cell apoptosis, and induced cell cycle arrest, potentially by regulating the ARF1/ERK signaling pathway. These results suggest the role of TMED2 as an oncogene in the progression of OSCC and highlight its potential as a therapeutic target to treat OSCC.
